# Aberrant expressions of circulating lncRNA NEAT1 and microRNA‐125a are linked with Th2 cells and symptom severity in pediatric allergic rhinitis

**DOI:** 10.1002/jcla.24235

**Published:** 2022-01-22

**Authors:** Xionghui Wu, Sijun Zhao, Weiqing Huang, Lihua Huang, Min Huang, Xinyou Luo, Shuting Chang

**Affiliations:** ^1^ Department of Otorhinolaryngology Head and Neck Surgery Hunan Children's Hospital Changsha China; ^2^ Department of Neonatology Hunan Children's Hospital Changsha China; ^3^ Laboratory for Medical Center The Third Xiangya Hospital of Central South University Changsha China

**Keywords:** allergic rhinitis, disease risk and severity, long noncoding RNA nuclear enriched abundant transcript 1, microRNA‐125a, Th1/Th2 imbalance

## Abstract

**Objective:**

Long noncoding RNA nuclear enriched abundant transcript 1 (lnc‐NEAT1) and its target microRNA‐125a (miR‐125a) are reported to regulate immune and inflammation process in allergic rhinitis (AR). Hence, this study intended to investigate the correlation between lnc‐NEAT1 and miR‐125a expressions, as well as their clinical values in pediatric AR patients.

**Methods:**

Peripheral blood mononuclear cell samples from 80 pediatric AR patients, 40 disease controls (DCs), and 40 healthy controls (HCs) were collected to detect lnc‐NEAT1 and miR‐125a expressions by reverse transcription‐quantitative polymerase chain reaction. For pediatric AR patients only, serum interferon‐gamma (IFN‐γ) and interleukin (IL)‐10 were measured by enzyme linked immunosorbent assay; meanwhile, T helper (Th) 1 and Th2 cells in CD4^+^ T cells were analyzed by flow cytometry.

**Results:**

Lnc‐NEAT1 was overexpressed, while miR‐125a downregulated in pediatric AR patients compared to DCs and HCs (all *p* < 0.001). Moreover, lnc‐NEAT1 expression negatively correlated with miR‐125a expression in pediatric AR patients (*p* = 0.002), but not in DCs (*p* = 0.226) or HCs (*p* = 0.237). Furthermore, in pediatric AR patients, lnc‐NEAT1 expression positively associated with TNSS (*p* < 0.001), sneezing score (*p* = 0.006), and congestion score (*p* = 0.008); miR‐125a expression was negatively related to TNSS (*p* < 0.001), itching score (*p* = 0.040), and sneezing score (*p* = 0.005). Additionally, lnc‐NEAT1 expression positively, while miR‐125a expression negatively correlated with Th2 cells and IL‐10 (all *p* < 0.05), but they were not correlated with Th1 cells or IFN‐γ in pediatric AR patients.

**Conclusion:**

Circulating lnc‐NEAT1 and miR‐125a are aberrantly expressed and linked with Th2 cells and symptom severity in pediatric allergic rhinitis.

## Graphical abstract

This study enrolled 80 pediatric AR patients, 40 DCs, and 40 HCs to detect their blood lnc‐NEAT1 and miR‐125a expression by RT‐qPCR. For pediatric AR patients only, serum IFN‐γ and IL‐10 were measured by ELISA; meanwhile Th1 and Th2 cells in CD4^+^ T cells were analyzed by flow cytometry. Intriguingly, lnc‐NEAT1 was overexpressed while miR‐125a downregulated in pediatric AR patients than in DCs and HCs. Moreover, lnc‐NEAT1 negatively correlated with miR‐125a in pediatric AR patients. Furthermore, in pediatric AR patients, lnc‐NEAT1 positively associated with TNSS, sneezing score and congestion score; miR‐125a was negatively related to TNSS, itching score and sneezing score. Additionally, lnc‐NEAT1 positively, while miR‐125a negatively correlated with Th2 cells and IL‐10 in pediatric AR patients.

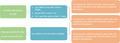

## 1 INTRODUCTION

Allergic rhinitis (AR) is a hypersensitivity reaction whose symptoms mainly include nasal itching, congestion, rhinorrhea, sneezing, etc., which is caused by immunoglobulin E (IgE)‐mediated inflammatory response when patients are exposed to allergens.^1^, ^2^ Remarkably, AR is the most prevalent allergic disease in the pediatric population; its prevalence ranges from 8.5% to 14.6% according to the International Study of Asthma and Allergies in Childhood (ISAAC).^3^, ^4^ Although there are various treatment strategies for pediatric allergic rhinitis, such as environmental controls, nasal anticholinergics, leukotriene receptor antagonists (LTRA), topical steroid, antihistamines, and allergen immunotherapy (AIT), recurrence of pediatric AR patients is still common because it is difficult to completely avoid the potential allergens.^5^, ^6^ Additionally, AR together with its comorbidities (including asthma, sinusitis, upper airway cough syndrome, and otitis media) further impairs the life quality, school performance, and sleeping quality of pediatric AR patients.^7^, ^8^, ^9^ Therefore, identifying biomarkers to assist the diagnosis of and the management for pediatric AR patients is necessary.

Long noncoding RNA nuclear enriched abundant transcript 1 (lnc‐NEAT1), located on chromatin 11, is a vital component of nuclear paraspeckles and implicated in the progression of many inflammation‐related diseases, such as sepsis and asthma.^10^, ^11^, ^12^, ^13^ For instance, lnc‐NEAT1 has pro‐inflammatory biological property and promotes the secretion of many inflammatory cytokines in asthma via activating multiple signaling pathways.^14^ Furthermore, some studies exhibit that lnc‐NEAT1 binding with microRNA‐125a (miR‐125a) participates in the pathogenesis of multiple human diseases (including multiple myeloma, acute ischemic stroke, and coronary artery disease).^15^, ^16^, ^17^ In terms of AR, although one study explores that lnc‐NEAT1 together with miR‐125a manifests close correlation with the disease risk of AR, it only surveys AR patients whose ages are above 18 years and collects their inferior turbinate mucosa, while as mentioned above, AR occurs in the pediatric population to a large extent, and it is inconvenient to collect their nasal mucosa under local anesthesia.^18^

Hence, this study enrolled 80 pediatric AR patients, 40 non‐allergic nasal pediatric patients, and 40 healthy pediatric subjects to detect their lnc‐NEAT1 and miR‐125a expressions in the peripheral blood, aiming to investigate the correlation of circulating lnc‐NEAT1 and miR‐125a with disease risk and severity of pediatric AR, also to explore their associations with T helper (Th)1, Th2 cells and their secreted cytokines in the pediatric AR patients.

## 2 MATERIALS AND METHODS

### 2.1 Subjects

With the approval of the Institutional Research Ethics Committee, this study consecutively enrolled 80 pediatric AR patients with obvious symptoms were treated in our hospital from February 2020 to August 2020. The enrollment criteria were as follows: (1) diagnosed as AR according to Allergic Rhinitis and its Impact on Asthma (ARIA) guidelines, 2016 revision^19^; (2) less than 16 years old; (3) agreed to provide peripheral blood (PB) samples for the present study. The exclusion criteria were as follows: (1) complicated with chronic nasal diseases, such as chronic rhinosinusitis or nasal polyposis; (2) complicated with severe respiratory diseases; (3) presented as severe infections; (4) concomitant with malignancies, cancers, or autoimmune diseases. Besides, during the same period, another 40 pediatric patients with snore or other non‐allergic nasal diseases were treated in our hospital, as well as 40 healthy pediatric subjects who underwent physical examination in our hospital were enrolled as disease controls (DCs) and healthy controls (HCs), respectively. The exclusion criteria for AR patients were also appropriate for DCs and HCs. The guardians of the enrolled subjects signed the informed consent.

### 2.2 Collection of data and samples

After enrollment, the demographics of all subjects were recorded, and the serum IgE level of all subjects was examined. For pediatric AR patients, the disease severity was evaluated by the total nasal symptom score (TNSS) in accordance with a previous study,^20^ which included the nasal rhinorrhea score, the itching score, the sneezing score, and the congestion score. Meanwhile, PB samples were collected from pediatric AR patients on admission and from DCs and HCs after enrollment, respectively. Then, the peripheral blood mononuclear cell (PBMC) was isolated from PB samples of all subjects by the gradient density centrifugation, and serum was separated from PB samples of pediatric AR patients as well.

### 2.3 Determination of lnc‐NEAT1 and miR‐125a expressions

The lnc‐NEAT1 and miR‐125a expressions in PBMC of all subjects were detected using the reverse transcription‐quantitative polymerase chain reaction (RT‐qPCR) assay. Total RNA was extracted by RNeasy Protect Mini Kit (Qiagen), then reserve transcription was completed using PrimeScript™ RT reagent Kit (Takara). After that, qPCR was achieved by KOD SYBR^®^ qPCR Mix (TOYOBO). The relative expressions of lnc‐NEAT1 and miR‐125a were calculated using the 2^−ΔΔ^ *^C^* ^t^ method. GAPDH was used as the internal reference for lnc‐NEAT1, and U6 as the internal reference for miR‐125a. Besides, qPCR primers were designed referring to previous studies.^21^, ^22^

### 2.4 Determination of Th1 and Th2 cell levels

The proportions of Th1 and Th2 cells in CD4^+^ T cells of pediatric AR patients were analyzed by the multicolor flow cytometric analysis using commercial Human Cell Differentiation Kit (Bio‐Techne China Co. Ltd.). The procedure of the experiment was performed strictly according to the kit instruction. In brief, after stimulation, T helper cells were stained using specific fluorescent antibodies, then the cells were counted by a fluorescence activated cell sorting (FACS) flow cytometer using anti‐human interferon‐gamma (IFN‐γ) antibody and anti‐human interleukin 4 antibody (Bio‐Techne China Co. Ltd.). Sequentially, the proportions of Th1 and Th2 cells in CD4^+^ T cells were calculated.

### 2.5 Determination of IFN‐γ and IL‐10

IFN‐γ (Th1 cell cytokine) and IL‐10 in serum of pediatric AR patients were detected by enzyme linked immunosorbent assay (ELISA), which was implemented using commercial Human ELISA Kit (Bio‐Techne China Co. Ltd.). The experiment was performed referring to the specification provided by the manufacturer.

### 2.6 Statistical analysis

SPSS 24.0 (IBM Corp.) and GraphPad Prism 6.01 (GraphPad Software Inc.) were used for statistical analysis and graph construction, respectively. Comparisons of clinical characteristics among groups were analyzed by one‐way analysis of variance (ANOVA) test, chi‐square test, and Kruskal‐Wallis H rank sum test. Comparisons of lnc‐NEAT1 and miR‐125a expressions among groups were determined by Kruskal‐Wallis H rank‐sum test and adjusted by the Bonferroni test. The receiver operating characteristic (ROC) curve analysis and the area under the curve (AUC) were used to evaluate the performance of lnc‐NEAT1 and miR‐125a expressions, and lnc‐NEAT1/miR‐125a axis in identifying different subjects. Correlations of two continuous variables were assessed using Spearman's rank correlation test. Statistical significance was concluded when *P* value <0.05 was indicated in the above analysis.

## 3 RESULTS

### 3.1 Clinical characteristics

The mean ages of pediatric AR patients, DCs, and HCs were 6.2 ± 2.7 years, 6.7 ± 2.5 years, and 6.5 ± 3.1 years, respectively (Table 1). Meanwhile, there were 42 (52.5%) males and 38 (47.5%) females in the AR group, 16 (40.0%) males and 24 (60.0%) females in DCs group, as well as 19 (47.5%) males and 21 (52.5%) females in HCs group. Additionally, the serum IgE of pediatric AR patients, DCs and HCs were 308.2 (182.4–496.4) IU/ml, 26.7 (17.3–44.0) IU/ml and 20.8 (15.2–30.7) IU/ml, accordingly. Besides, the TNSS of pediatric AR patients was 7.8 ± 1.8. Most clinical characteristics were of no difference including age (*p *= 0.582), gender (*p *= 0.432), height (*p *= 0.675), and weight (*p *= 0.846), except the IgE level, which was varied (*p *< 0.001) among all subjects. Moreover, the detailed clinical characteristics are listed in Table 1.

**TABLE 1:** Clinical characteristics

Items	HCs (*N* = 40)	DCs (*N* = 40)	AR (*N* = 80)	*p* Value
Age (years), mean ± SD	6.5 ± 3.1	6.7 ± 2.5	6.2 ± 2.7	0.582
Gender, No. (%)				0.432
Male	19 (47.5)	16 (40.0)	42 (52.5)	
Female	21 (52.5)	24 (60.0)	38 (47.5)	
Height (cm), mean ± SD	118.7 ± 17.8	119.8 ± 16.1	117.0 ± 17.2	0.675
Weight (kg), mean ± SD	23.9 ± 8.0	23.9 ± 8.1	23.1 ± 8.7	0.846
Serum IgE (IU/ml), median (IQR)	20.8 (15.2–30.7)	26.7 (17.3–44.0)	308.2 (182.4–496.4)	<0.001
TNSS, mean ± SD	–	–	7.8 ± 1.8	–
Nasal rhinorrhea score, mean ± SD	–	–	2.0 ± 0.8	–
Itching score, mean ± SD	–	–	1.9 ± 0.8	–
Sneezing score, mean ± SD	–	–	2.0 ± 0.9	–
Congestion score, mean ± SD	–	–	1.9 ± 0.9	–

Note

AR, allergic rhinitis; DCs, disease controls; HCs, healthy controls; IgE, immunoglobulin E; IQR, interquartile range; SD, standard deviation; TNSS, total nasal symptom score.

### 3.2 Expressions of lnc‐NEAT1 and miR‐125a in pediatric AR patients, DCs, and HCs

**FIGURE 1:**
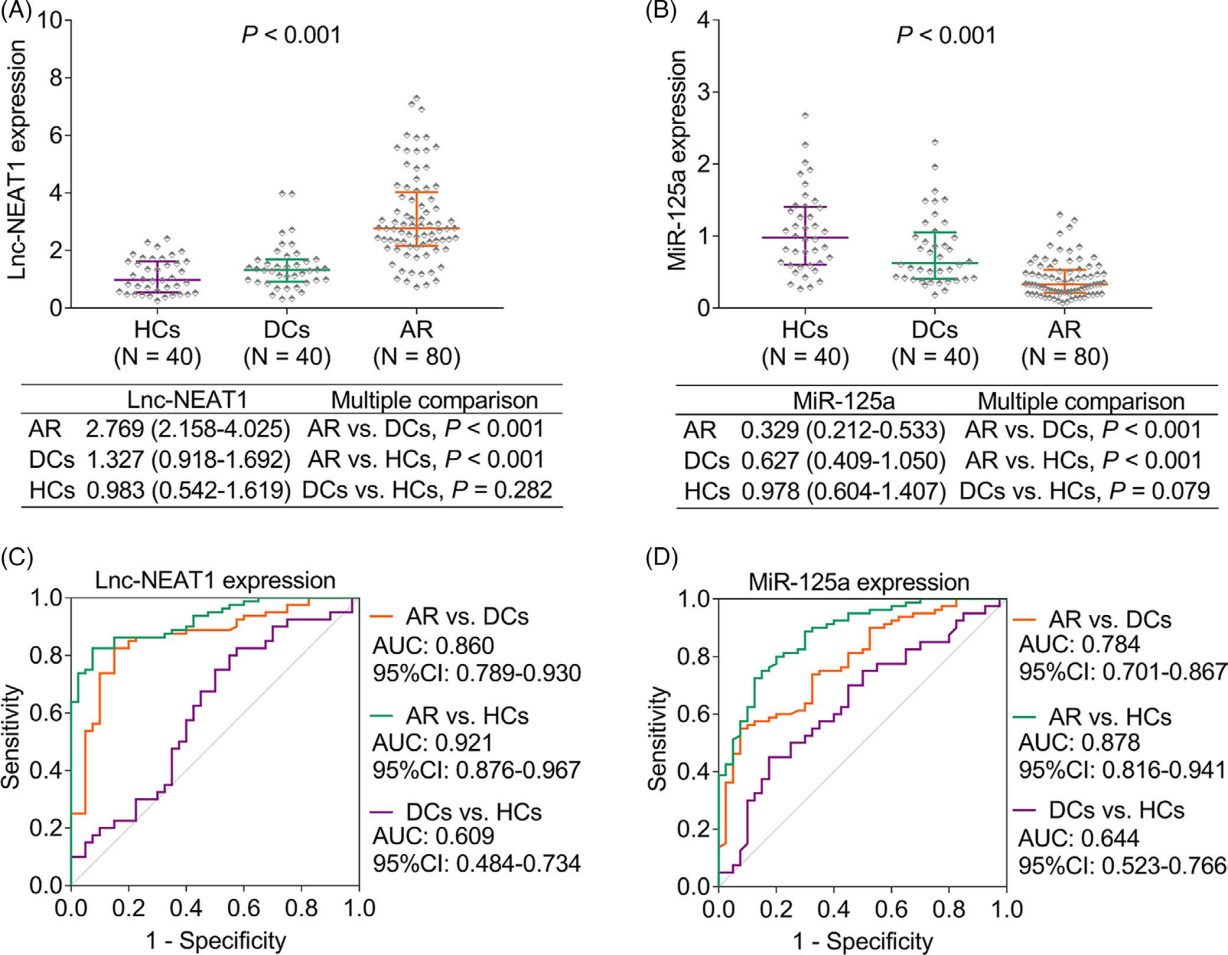
Different expressions of lnc‐NEAT1 and miR‐125a among pediatric AR patients, DCs, and HCs. Comparison of lnc‐NEAT1 (A) and miR‐125a (B) expressions among pediatric AR patients, DCs, and HCs; The value of lnc‐NEAT1 (C) and miR‐125a (D) in distinguishing different subjects

The expressions of lnc‐NEAT1 (*p *< 0.001) and miR‐125a (*p *< 0.001) were both differed among pediatric AR patients, DCs, and HCs (Figure 1A,B). In detail, the lnc‐NEAT1 expression was highest in the pediatric AR patients, followed by DCs, and the lowest in HCs. Meanwhile, the miR‐125a expression was the top in HCs, the second high in DCs, and the lowest in pediatric AR patients.

Besides, the ROC curve disclosed that the lnc‐NEAT1 expression had good diagnosing value to distinguish pediatric AR patients from HCs (AUC: 0.921, 95% confidence interval (CI): 0.876–0.967) and pediatric AR patients from DCs (AUC: 0.860, 95% CI: 0.789–0.930) (Figure 1C). Likewise, the miR‐125a expression was also of good value to discriminate pediatric AR patients from HCs (AUC: 0.878, 95% CI: 0.816–0.941) and DCs (AUC: 0.784, 95% CI: 0.701–0.867; Figure 1D).

As to the lnc‐NEAT1/miR‐125a axis, it also differed among pediatric AR patients, DCs, and HCs (*p *< 0.001, Figure S1A), which could differentiate pediatric AR patients from HCs (AUC: 0.954, 95% CI: 0.916–0.992) and pediatric AR patients from DCs (AUC: 0.874, 95% CI: 0.813–0.934, Figure S1B).

### 3.3 Correlation of lnc‐NEAT1 with miR‐125a in pediatric AR patients

**FIGURE 2:**
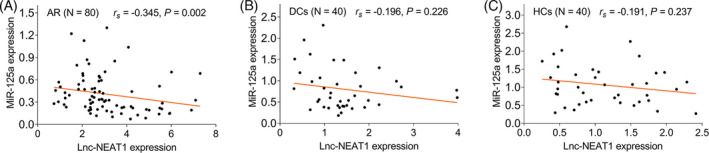
Lnc‐NEAT1 negatively correlated with miR‐125a in pediatric AR patients, but not in DCs or in HCs. Correlation of lnc‐NEAT1 expression with miR‐125a in pediatric AR patients (A), DCs (B), and HCs (C)

In pediatric AR patients, the lnc‐NEAT1 expression was negatively correlated with the miR‐125a expression (*r_s_* = −0.345, *p *= 0.002), while no correlation was found in the lnc‐NEAT1 expression with the miR‐125a expression in DCs (*r_s_* = −0.196, *p *= 0.226) or in HCs (*r_s_* = −0.191, *p *= 0.237; Figure 2A–C).

### 3.4 Correlation of lnc‐NEAT1 and miR‐125a with TNSS and its subitems in pediatric AR patients

**FIGURE 3:**
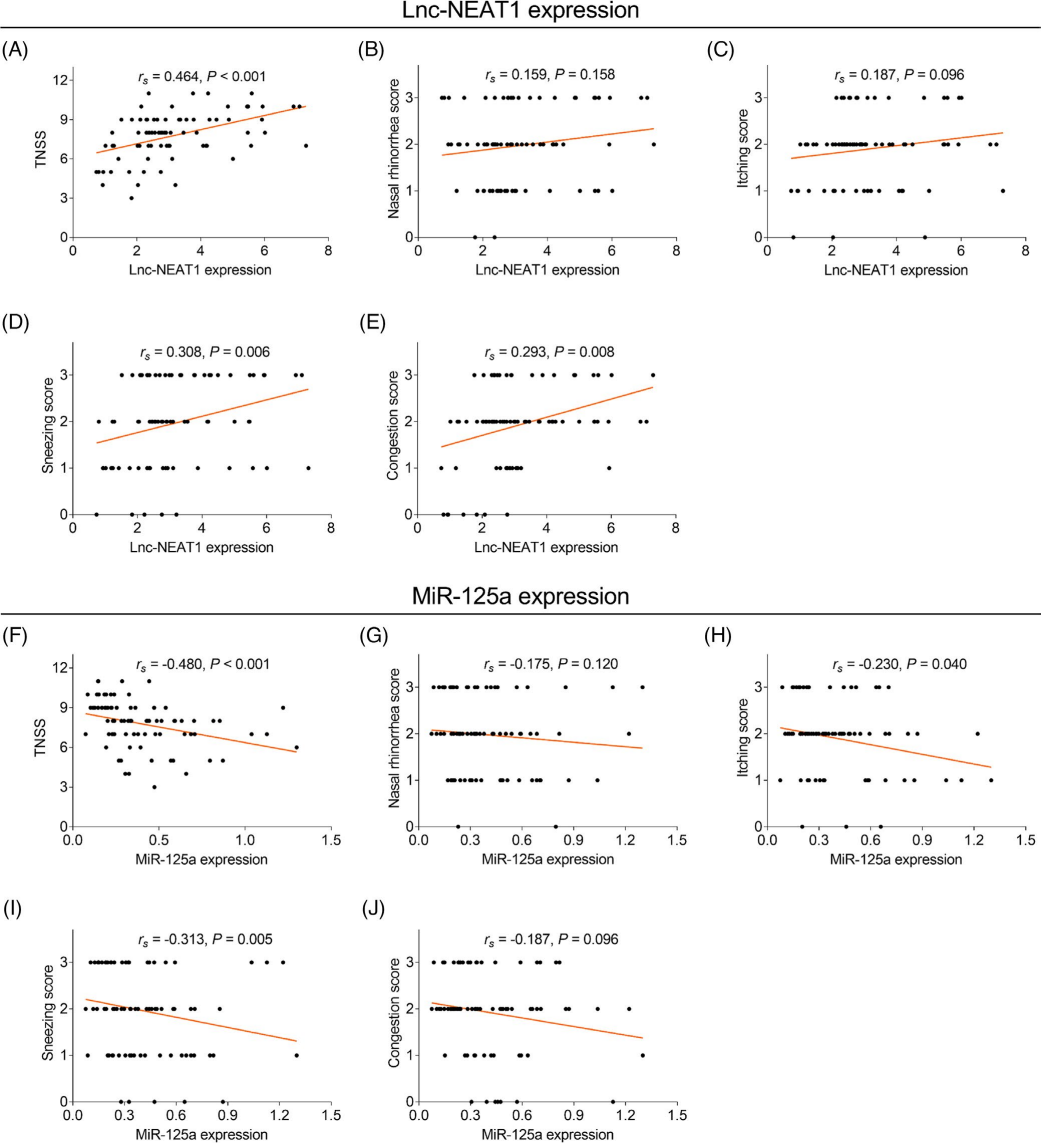
Lnc‐NEAT1 positively, while miR‐125a negatively associated with AR patients’ symptoms. Correlation of lnc‐NEAT1 with TNSS (A), nasal rhinorrhea score (B), itching score (C), sneezing score (D), and congestion score (E); Correlation of miR‐125a with TNSS (F), nasal rhinorrhea score (G), itching score (H), sneezing score, (I) and congestion score (J) in pediatric AR patients

The lnc‐NEAT1 expression was positively associated with the TNSS (*r_s_* = 0.464, *p *< 0.001), the sneezing score (*r_s_* = 0.308, *p* = 0.006), and the congestion score (*r_s_* = 0.293, *p* = 0.008), while no association was observed in the lnc‐NEAT1 expression with the nasal rhinorrhea score (*r_s_* = 0.159, *p* = 0.158) and the itching score (*r_s_* = 0.187, *p* = 0.096; Figure 3A–E). Moreover, the miR‐125a expression was negatively related to the TNSS (*r_s_* = −0.480, *p *< 0.001), the itching score (*r_s_* = −0.230, *p* = 0.040), and the sneezing score (*r_s_* = −0.313, *p* = 0.005); nevertheless, there was no correlation of the miR‐125a expression with the nasal rhinorrhea score (*r_s_* = −0.175, *p* = 0.120) or the congestion score (*r_s_* = −0.187, *p* = 0.096; Figure 3F–J).

### 3.5 Correlation of lnc‐NEAT1 and miR‐125a with Th1 and Th2 cells, as well as their secreted cytokines in the pediatric AR patients

**FIGURE 4:**
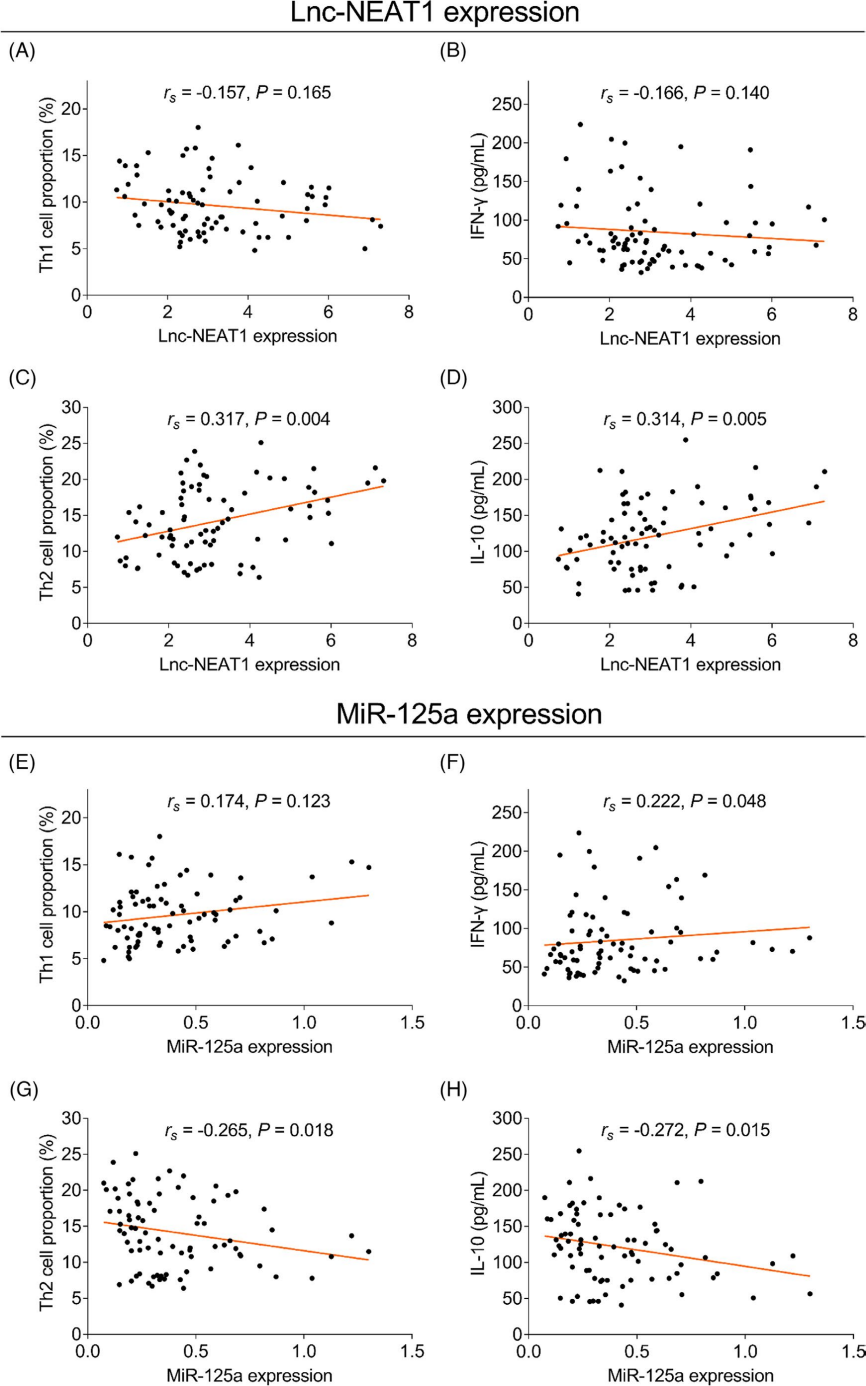
Lnc‐NEAT1 positively, while miR‐125a negatively correlated with Th2 cells and secreted cytokines in pediatric AR patients. Correlation of lnc‐NEAT1 with Th1 cells (A), IFN‐γ (B), Th2 cells (C) and IL‐10 (D) in pediatric AR patients; Correlation of miR‐125a with Th1 cells (E), IFN‐γ (F), Th2 cells (G), and IL‐10 (H) in pediatric AR patients

The lnc‐NEAT1 expression was positively correlated with Th2 cells (*r_s_* = 0.317, *p* = 0.004) and IL‐10 (*r_s_* = 0.314, *p* = 0.005), but not correlated with Th1 cells (*r_s_* = −0.157, *p* = 0.165) or IFN‐γ (*r_s_* = −0.166, *p* = 0.140; Figure 4A–D). In terms of the miR‐125a expression, it was negatively associated with Th2 cells (*r_s_* = −0.265, *p* = 0.018) and IL‐10 (*r_s_* = −0.272, *p* = 0.015), but there was no relationship between the miR‐125a expression and Th1 cells (*r_s_* = 0.174, *p* = 0.123); besides, the miR‐125a expression was positively correlated with IFN‐γ (*r_s_* = 0.222, *p* = 0.048) (Figure 4E–H).

## 4 DISCUSSION

In this study, we found that: (1) Lnc‐NEAT1 was overexpressed, while its target miR‐125a was downregulated in pediatric AR patients compared to DCs and HCs. Additionally, both the lnc‐NEAT1 expression and the miR‐125a expression suggested good potential in distinguishing pediatric AR patients from DCs and HCs. (2) The lnc‐NEAT1 expression was negatively correlated with the miR‐125a expression in pediatric AR patients. (3) In pediatric AR patients, the lnc‐NEAT1 expression was positively related to disease severity, while the elevated miR‐125a expression was linked with alleviative disease severity. (4) In pediatric AR patients, the lnc‐NEAT1 expression was positively related to Th2 cells and IL‐10, but not correlated with Th1 cells or IFN‐γ, which caused Th1/Th2 imbalance.

Lately, some studies have explored that lnc‐NEAT1 and miR‐125a are dysregulated in some inflammatory disorders, such as bronchial asthma and AR.^14^, ^16^, ^18^, ^23^ For instance, one study collects inferior turbinate mucosa and finds that lnc‐NEAT1 is dysregulated, which can distinguish adult AR patients from controls.^18^ However, the diagnostic value of lnc‐NEAT1 and miR‐125a in pediatric AR patients remains unclear. Regarding that, since it was infeasible to collect nasal mucosa samples from children, this study detected blood lnc‐NEAT1 and miR‐125a expressions. Subsequently, we discovered that the lnc‐NEAT1 expression was upregulated, while the miR‐125a expression was downregulated in pediatric AR patients compared with DCs and HCs; meanwhile, both of them could identify AR risk, which was partially in line with the study mentioned above.^18^ The probable reasons might be that (1) the lnc‐NEAT1 expression promoted Th2 cells activation and further exacerbated the imbalance between Th1 and Th2 cells.^24^ Moreover, the Th1/Th2 imbalance was the leading cause of increased AR risk.^25^ Hence, the lnc‐NEAT1 expression presented a good identifying value for pediatric AR risk. (2) The lnc‐NEAT1 expression was positively related to several inflammatory cytokines (including IL‐1β and IL‐6), which were reported to be increased in the AR patients than in controls.^14^, ^18^, ^26^, ^27^ Therefore, lnc‐NEAT1 was overexpressed in pediatric AR patients than in DCs and HCs. (3) In terms of miR‐125a, it could also regulate the homeostasis of Th1 and Th2 cells as well as suppress inflammation via stabilizing signal transducer and activator of transcription 3 (STAT3) activity and inhibiting immune responses.^28^ Thus, the miR‐125a expression was downregulated in pediatric AR patients compared with DCs and HCs.

Apart from exploring the dysregulation of lnc‐NEAT1 and miR‐125a, various studies also note that they are inter‐correlated in sepsis, coronary artery disease, etc.^16^, ^29^ In line with previous studies, our study also revealed a negative correlation was found between circulating lnc‐NEAT1 and miR‐125a in the pediatric AR patients, but not in DCs or HCs, which could be explained by that in healthy subjects or non‐hyperimmune diseases, lnc‐NEAT1 and miR‐125a expressions were in the state of immune homeostasis and neither of them was dysregulated, while the occurrence of AR might cause excessive imbalance between lnc‐NEAT1 and miR‐125a and further resulted in the enhanced negative correlation between them.^30^ Thus, the lnc‐NEAT1 expression was negatively correlated with the miR‐125a expression in the pediatric AR patients, while no correlation was observed between the lnc‐NEAT1 and miR‐125a expressions in DCs or HCs.

Recent evidence indicates that the lnc‐NEAT1 expression is positively associated with disease severity in asthma patients and other allergic diseases.^14^ Analogously, in the current study, we found that circulating lnc‐NEAT1 was positively correlated with disease severity in pediatric AR patients, while the miR‐125a expression was negatively associated with AR severity. The possible explanations might be that (1) lnc‐NEAT1 promoted the secretion of several inflammasomes (including NLR‐family CARD‐containing protein 4 (NLRC4), absent in melanoma 2 (Aim2), and NOD‐like receptor family pyrin domain containing 3 (NLRP3), etc.), then aggravated the inflammation.^14^, ^31^, ^32^ Meanwhile, the severity of AR was positively related to IgE‐mediated inflammation, therefore, elevated lnc‐NEAT1 expression was associated with deteriorated disease severity in pediatric AR patients.^33^ (2) Lnc‐NEAT1 might cause smooth muscle contraction and mucus secretion through releasing histamine to decrease epithelial barrier, then led to more severe allergic symptoms.^18^, ^34^, ^35^ As a result, the lnc‐NEAT1 expression was positively linked with the disease severity of pediatric AR patients. (3) MiR‐125a suppressed the polarization of macrophages toward M1, then inactivated allergic inflammatory responses.^36^ Hence, the miR‐125a expression was negatively correlated with AR severity. Moreover, it was reported that lnc‐NEAT1 might facilitate the polarization of macrophages toward M2 through Janus kinase 2 (JAK2)/STAT3 signaling pathway, while M2 macrophage was positively related to AR severity.^37^, ^38^ Therefore, the lnc‐NEAT1 expression was positively linked with the disease severity of pediatric AR patients. (4) The lnc‐NEAT1 expression might be related to exacerbated allergic response via inducing the secretion of pro‐inflammatory cytokines and stimulating inflammatory responses; thus, the lnc‐NEAT1 expression was positively related to the disease severity of pediatric AR patients.^14^, ^39^

AR is also exacerbated due to the imbalance of Th1/Th2 differentiation, and its imbalance arouses the binding of IgE with eosinophil and mast cells, which is a promoting factor of allergic inflammation.^40^, ^41^, ^42^, ^43^ However, the correlation of the lnc‐NEAT1 and miR‐125a expressions with Th1/Th2 imbalance in pediatric AR patients remains unanswered. This study disclosed that in pediatric AR patients, circulating lnc‐NEAT1 was positively related to Th2 cells and IL‐10, but not correlated with Th1 cells or IFN‐γ, which caused Th1/Th2 imbalance in the pediatric AR patients. The possible reasons to explain these results were as follows: (1) Lnc‐NEAT1 promoted the signal transducer and activator of transcription 6 (STAT6) expression in CD4^+^ T cells; moreover, STAT6 signaling pathway induced the differentiation of T cells toward Th2 cells.^24^ Hence, the lnc‐NEAT1 expression was positively correlated with Th2 cells. (2) Lnc‐NEAT1 probably mainly enhanced the differentiation of T cells into Th2 cells, and the proportion of Th1 cells was subsequently indirectly affected; therefore, the lnc‐NEAT1 expression was positively associated with Th2 cells, but not correlated with Th1 cells.

There were some limitations in this study. Firstly, the sample size was relatively small, which might cause a weak statistical power, and a further study with a larger sample size was necessary. Secondly, the lnc‐NEAT1 and miR‐125a expressions were detected at a single time point in this current study; therefore, their longitudinal change for the continuous monitoring of the disease progression needed to be explored. Thirdly, we hypothesized that the lncRNA NEAT1 expression involved in the allergic inflammation response of AR via targeting miR‐125a, whereas its detailed molecular mechanisms needed to be answered in the future study. Fourthly, according to the type of allergen, AR could be divided into seasonal AR and perennial AR; and according to the frequency of symptoms, AR could be classified into intermittent AR and persistent AR.^44^ Thus, the correlation of lnc‐NEAT1 and miR‐125a expressions with AR subtypes could be explored in the future studies. Fifthly, the original of lnc‐NEAT1 and miR‐125a was still unclear, which deserved further investigation. Sixthly, IL‐4 was the main Th2‐secreted cytokine, while the corresponding data were missed in the current study.

In conclusion, circulating lnc‐NEAT1 and miR‐125a are dysregulated and correlated with the disease severity and Th1/Th2 imbalance in the pediatric AR patients, which makes it possible to distinguish the risk stratification and consequently help the clinicians provide individual treatment to each pediatric AR patient. Hence, they may serve as potential biomarkers for the management of the pediatric AR patients.

## CONFLICT OF INTEREST

The authors declare that they have no competing interests.

## Supporting information

Fig S1

## Data Availability

Data sharing is not applicable to this article as no datasets were generated or analyzed during the current study.
