# Supplementary figures and images for: Aberrant expressions of circulating lncRNA NEAT1 and microRNA‐125a are linked with Th2 cells and symptom severity in pediatric allergic rhinitis
Source: J Clin Lab Anal. 2022 Jan 22;36(3):e24235. doi: 10.1002/jcla.24235 (PMC8906029; doi:10.1002/jcla.24235)

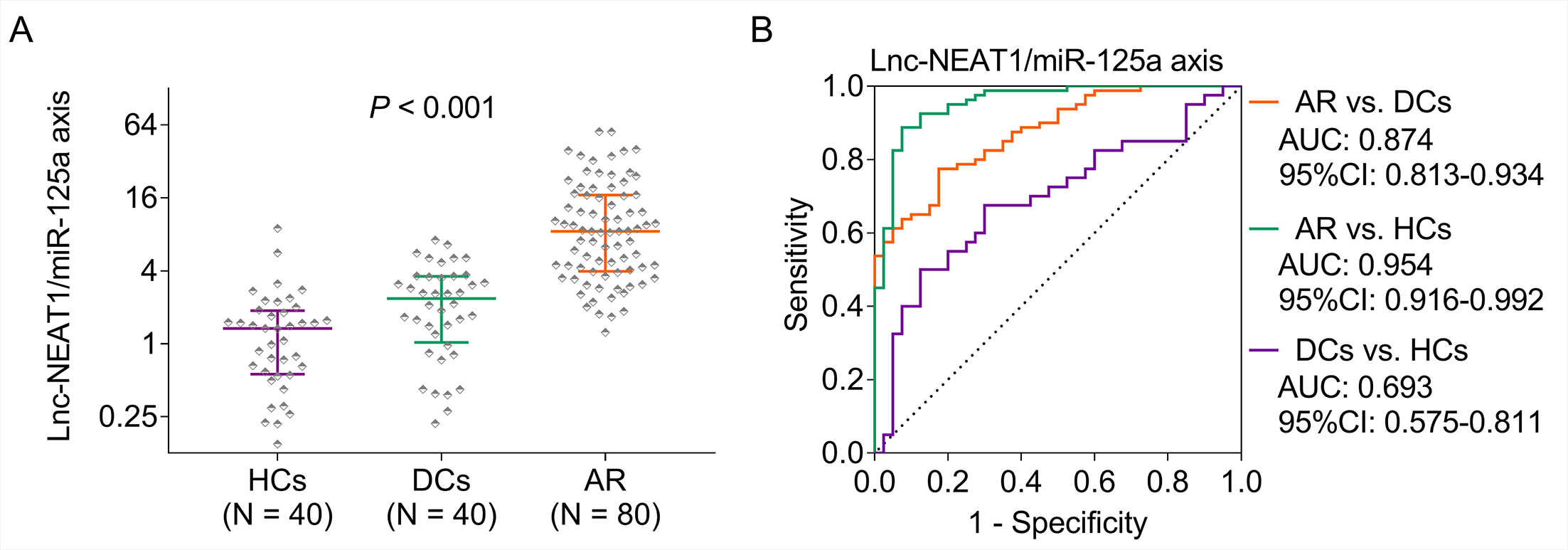

Supplement: Supplementary file 1 — Fig S1 [file JCLA-36-e24235-s001.tif]
